# Cross-Sectional Analysis of Canadian Anesthesiology Residency Program Website Content

**DOI:** 10.7759/cureus.23410

**Published:** 2022-03-22

**Authors:** Amolpreet S Toor, Denise J Wooding, Sarmad Masud, Faisal Khosa

**Affiliations:** 1 Medicine, University of British Columbia, Vancouver, CAN; 2 Anesthesiology, Shalamar Medical and Dental College and Hospital, Lahore, PAK; 3 Radiology, Vancouver General Hospital, Vancouver, CAN

**Keywords:** online content, website comprehensiveness, residency programs, program websites, anesthesiology

## Abstract

Background

Residency program websites are an important resource widely used by prospective applicants when applying to programs. The objectives of our study were to evaluate the program content available on Canadian anesthesiology residency program websites using established criteria and identify any areas for improvement.

Methods

In this cross-sectional study, we evaluated the content available on accredited anesthesiology residency training program websites, between July and August 2021, using 54 criteria provided in the following domains: recruitment; faculty; residents; education and research; clinical work; incentives; wellness; and environment. Website scores were analyzed using descriptive statistics and presented as median (interquartile range), percentage (%), and range.

Results

We identified 17 programs with publicly available functional websites. Overall, residency programs met a median of 28 (interquartile range: 18-36) website criteria out of 54 (51.9%). Education and research was the highest-scoring domain among residency programs (median 77.8% of criteria met), while resident information and incentives were the lowest (14.3%).

Conclusion

Canadian anesthesiology residency program websites include information on many domains relevant to prospective applicants, including education and research. However, most websites require improvement and content updates for faculty information, resident information, incentives, wellness, and environment.

## Introduction

The process of choosing and applying for residency programs is challenging for medical students [1-3]. Online content available on anesthesiology residency websites is a widely used resource for obtaining program information among prospective applicants [1,4]. Many studies have shown that the quality and content of post-graduate medical program websites are important factors in making an informed decision for an application [4-7]. For instance, an investigation amongst the United States (US) anesthesia residency applicants demonstrated that 98% of applicants explored program websites during the application process, and 56% reported that the website content influenced their decision to apply to a program [4]. Moreover, the COVID-19 pandemic has only increased the reliance on virtual platforms for medical trainee recruitment, making program websites a major avenue to communicate program-specific information [8-10]. Despite this, postgraduate medical program websites across many specialties (e.g., obstetrics and gynecology, and radiology) lack important program content that is valued by prospective applicants [11-14].

To better facilitate applicant decision-making, it is pertinent to maintain a resource of comprehensive information for prospective applicants. This will help both medical trainees and program recruitment committees to find the most suitable match for their respective goals, aims, and needs. Accordingly, the objectives of our study were to: (1) assess the content available to prospective applicants on Canadian anesthesiology residency websites using established criteria [4,5,7,11-14]; (2) identify any content areas requiring improvement.

## Materials and methods

Study design

We conducted a cross-sectional study to evaluate program information available to applicants on Canadian anesthesiology residency training websites between July and August 2021. The study did not require research ethics board approval as publicly available data was evaluated (Tri-Council Policy Statement 2 Article 2.2). A 54-criteria system used to evaluate program websites was created using: (1) established criteria from previously published postgraduate medical program website evaluations [4,5,7,11-14]; (2) criteria relevant to anesthesiology training (e.g., simulation training) according to the Royal College of Physicians and Surgeons of Canada (RCPSC) anesthesiology program standards [15-17]. The 54 criteria were organized into the following domains: recruitment, faculty information, resident information, education and research, clinical work, incentives, wellness, and environment (Table 1).

**Table 1:** Fifty-four-point criteria system used to evaluate Canadian anesthesiology residency program websites OSCE - Objective Structured Clinical Exam, AKT - Anesthesia Knowledge Test

Website criteria
Recruitment domain (8 criteria)
Introduction/welcome message
Program description
Program mission/vision/values
Program contact information (e.g., email and phone number)
Program accreditation
Number of residency positions
Admission process (e.g., application link, interview process)
Selection criteria
Faculty information domain (8 criteria)
Core faculty names
Faculty positions listed
Contact information (e.g., email)
Individual photos
Subspecialty listed
Educational background
Research/clinical interests
Research publications/presentations
Resident information domain (7 criteria)
Current resident contact (e.g., name, email)
Residency year
Individual photos
Educational background
Interests (e.g., academic and extracurricular interests)
Alumni contact (e.g., name, email)
Alumni career placements (e.g., fellowship, position, geographical location)
Education and research domain (9 criteria)
Curriculum/didactic learning (e.g., units, topics, schedule)
Assessments/evaluations (e.g., OSCE, AKT, simulation-based assessments)
Rounds (e.g., grand rounds, morbidity/mortality rounds)
Journal club
Research/scholarly opportunities
Past research (e.g., publications and presentations)
Professional development fund/grant opportunities
Academic time (e.g., academic days/half-days)
Educational resources (e.g., textbooks, online resources, library access)
Clinical work domain (8 criteria)
Clinical sites
Core rotation structure
Optional electives and rotations (e.g., off-service, out of province, international)
Caseload (e.g., cases per year)
Call schedule
Procedures/clinical skills (e.g., neuraxial block, endotracheal intubation)
Equipment mentioned (e.g., ultrasound)
Simulation training (e.g., crisis management)
Incentives domain (7 criteria)
Benefits (e.g., disability and health insurance)
Salary (numerical value)
Vacation
Statutory and floating holidays
Leave (e.g., educational, pregnancy, parental)
Moonlighting
Mentorship opportunities (e.g., academic, clinical, and peer mentorship)
Wellness domain (3 criteria)
Mental health/spiritual resources
Physical wellness activities/resources (e.g., fitness, sleep, nutrition)
Social wellness (e.g., program social events)
Environment domain (4 criteria)
Location highlights (e.g., city/neighborhood amenities, restaurants)
Local activities (e.g., outdoor activities)
Housing and living (e.g., cost of living)
Hospitals

Data collection

A list of RCPSC accredited anesthesiology residency programs was obtained from the Canadian Residency Matching System (CaRMS) website (https://www.carms.ca/match/r-1-main-residency-match/program-descriptions/). Anesthesiology Clinician Investigator Programs (CIP) were excluded from this study because RCPSC training requirements and goals for CIP differ from regular stream anesthesiology residency programs; it would not be appropriate to evaluate Anesthesiology CIP website content using our 54-criteria system (Table 2) [18].

**Table 2:** List of evaluated anesthesiology residency program websites

Anesthesiology residency program	Program website
University of British Columbia	https://bit.ly/3kz5jHD
University of Calgary	https://bit.ly/3ixflXk
University of Alberta	https://bit.ly/3zfYm2c
University of Saskatchewan	https://bit.ly/3ezMjVy
University of Manitoba	https://bit.ly/2UY9pOC
Northern Ontario School of Medicine	https://bit.ly/2UXrwEu
Western University	https://bit.ly/3Bip0th
McMaster University	https://bit.ly/2UVQnJ1
University of Toronto	https://bit.ly/3wOl3c7
Queen’s University	https://bit.ly/3hRQT3q
uOttawa	https://bit.ly/3rmQ8mj
McGill	https://bit.ly/3z9p3pa
Université de Montréal	https://bit.ly/2UplYmc
Université de Sherbrooke	https://bit.ly/3zbHObG
Université Laval	https://bit.ly/3rl4Aep
Dalhousie University	https://bit.ly/3hPATiL
Memorial University	https://bit.ly/2UXtOU6

Two website reviewers (AST and DJW) were trained to use the 54-criteria system by examining three randomly selected program websites. Discrepancies in scores were discussed, and coding was determined as a team to establish interrater reliability. Information was considered present on a program website if it was available on: (1) the program website or subpages; (2) linked documents (e.g., program brochures); or (3) websites hyperlinked on the official page (e.g., link to hospital websites). A score of one was recorded for each criterion met by a program website, and zero for every criterion absent. The trained reviewers independently analyzed each anesthesiology program website; the order of program websites for each reviewer was randomly selected using an online generator (https://www.random.org/lists/). The reviewers were not blinded because they were viewing the actual website [11,19].

Statistical analysis

All data were cataloged on Excel (version 16.53 for Mac; Microsoft, Redmond, WA). Website scores were analyzed using descriptive statistics and presented as median (interquartile range), percentage (%), and range. Statistical analyses were performed using SPSS Statistics (version 28 for Mac; IBM, Armonk, NY).

## Results

There are a total of 17 RCPSC accredited anesthesiology residency programs; all programs had a functional program website. Overall, the 17 residency program websites met a median of 28 (interquartile range: 18-36) website criteria out of 54 total criteria (51.9%) with scores ranging from 14 to 44 (Table 3).

**Table 3:** Descriptive statistics for website criteria met by Canadian anesthesiology residency programs IQR - interquartile range, n - number of criteria per domain

Domain	Median scores (IQR)	Median criteria met (%)	Range of scores
Recruitment (n = 8)	6 (5-7)	75.0	4-8
Faculty information (n = 8)	3 (2-8)	37.5	0-8
Resident information (n = 7)	1 (0-2)	14.3	0-5
Education and research (n = 9)	7 (6-8)	77.8	3-9
Clinical work (n = 8)	5 (3-6)	62.5	1-8
Incentives (n = 7)	1 (0-3)	14.3	0-5
Wellness (n = 3)	1 (0-3)	33.3	0-3
Environment (n = 4)	1 (1-3)	25.0	0-4
Overall (n = 54)	28 (18-36)	51.9	14-44

Within the highest-scoring domain (education and research), research opportunities were the most prevalent criterion present on program websites (17/17, 100%) followed by curriculum/didactic learning (14/17, 82.4%) and assessments/evaluations (14/17, 82.4%). Resident information and incentives were the lowest-scoring domains among residency websites (Table 3). Specifically, educational background, interests, and alumni names were the least prevalent criteria met by programs within the resident domain (1/17, 5.9%). Within the incentive’s domain, salary and moonlighting were the least prevalent criteria met by programs (1/17, 5.9%).

## Discussion

The purpose of our study was to assess program content available to prospective applicants on Canadian anesthesiology residency websites using a 54-point criteria system. Education and research were the highest-scoring domain among residency programs, while resident information and incentives were the lowest.

Residency websites effectively provided program information regarding research opportunities (17/17, 100%), curriculum/didactic learning (14/17, 82.4%), and assessments/evaluations (14/17, 82.4%) within the education and research domain. Similarly, a study of obstetrics and gynecology program websites in Canada and general surgery residency websites in the United States (US) and Puerto Rico found that research opportunities and didactic learning were prevalent criteria on residency program websites [11,20]. Additionally, an evaluation of 131 anesthesia residency program websites in the US reported that research program information was found in 88% (117/131) of programs, and educational program schedules were found in 67% (88/131) [4].

Information about faculty, residents, incentives, wellness, and environment were often omitted from anesthesia residency websites. Faculty and resident information (e.g., biography and interests) are considered important content areas on program websites among applicants [6]. Faculty clinical and research interests may give applicants insight on potential mentors and active research areas as part of a program. According to a recent US National Resident Matching Program (NRMP) survey conducted among residency applicants, perceived goodness of fit (e.g., the atmosphere, culture, feel of a program) was considered important in deciding application and program rank by 90.5% of US anesthesiology applicants [21]. Faculty interests, resident biographies, and extracurricular information may help applicants subjectively assess their perceived goodness of fit with a program, making these website criteria useful in recruiting prospective trainees [22]. Additionally, faculty and resident contact information provides an opportunity for applicants to further investigate a program and connect with current residents to learn about their personal experiences with a program [1,14]. However, it is possible that contact information may be omitted from websites for privacy or security purposes. Additionally, programs may rely on alternative avenues to provide faculty and resident information, including social events (e.g., meet and greets) and information sessions.

Information regarding wellness (e.g., mental, physical, social wellbeing resources) and incentives (e.g., benefits, salary, moonlighting) were also among the least commonly met criteria on anesthesia residency websites. This is despite the increasing emphasis of the anesthesiology specialty on work-life balance and burnout avoidance [18,23,24]. Work-life balance is a motivating factor among medical trainees when making career choices [25]. Physical, psychological, and financial well-being affect physician job satisfaction and the quality of care they provide [23,26,27]. Moreover, a perceived lack of support at work among anesthesiologists is significantly associated with physician burnout [23]. It is unclear whether the lack of wellness information is due to an actual lack of programming, but this seems unlikely given that resident wellness support is an RCPSC accreditation requirement [16]. However, programs may not prioritize wellness information as much as residents or applicants. This may be due to a lack of faculty awareness surrounding the scope of the problem affecting resident wellness (e.g., burnout and depression) [28]. Nonetheless, providing wellness information through program websites may influence prospective residents’ program preferences, and may increase resident awareness and accessibility to wellness resources and satisfaction with a program [24]. Programs may offer wellness (e.g., mental, physical, and social) information on a page of their website in the following ways: (1) describing any active wellness curriculum offered by the program; (2) listing wellness resources and services offered by the program; (3) accessible links to local, provincial, and RCPSC wellness resources (https://www.royalcollege.ca/rcsite/documents/about/covid-19-wellness-resources-hp-e).

Similarly, information about incentives, including salary, benefits, and moonlighting may help applicants with financial planning. Medical school graduates often have significant educational debt, a factor commonly associated with depression, burnout, low quality of life, and decreased job satisfaction [26]. Salary and benefits information can be directly listed on program websites and continually updated as changes occur. Alternatively, an accessible link to CaRMS provincial salaries and benefits for postgraduate training may be provided (https://www.carms.ca/match/r-1-main-residency-match/salary/#1511459027032-06ec5e41-5301). Opportunities such as moonlighting, a practice permitted by the RCPSC, support trainees dealing with financial hardship, reduce emotional exhaustion, and improve quality of life [26,29]. It is important to note that while moonlighting is permitted by the RCPSC, there is a regional variation in regulatory policies and eligibility for moonlighting depending on the provincial regulatory body and program [30]. Given the program variability in policies and eligibility for moonlighting, we recommend programs attach a document listing their eligibility criteria and contact information for applicant inquiries. Efforts to promote the availability of website information regarding program incentives may increase interest from applicants, especially those with educational debt, and families to support. 

A multitude of reasons may explain the absence of information on residency websites. There is the possibility that programs may not recognize the changing trends for obtaining residency information [8]; programs may assume most applicants are obtaining information by word of mouth. Residency programs may outsource website maintenance to digital and marketing companies, which may not regularly consult physicians or faculty members. Lastly, residency programs may not consider it necessary to develop comprehensive websites given a large number of qualified applicants.

Limitations

Data collection took place between July and August 2021 and did not account for website updates that may have taken place after this study period. The 54 criteria used to assess each program website were adapted from previous studies and although they provide a standardized framework for assessing program websites, there is no one set of criteria that can reflect the values of all prospective applicants. Future studies investigating which criteria are important to postgraduate Canadian anesthesiology trainees may be helpful to create a more representative criteria list. Data collection by different individuals may lead to varying results due to the subjectivity involved in identifying the presence of information on program websites. Nevertheless, two trained investigators independently evaluated each program website to mitigate the potential for variability.

## Conclusions

In this cross-sectional study, we found that Canadian anesthesiology residency training program websites met most criteria in the education and research domain. However, many program websites lacked content pertinent to prospective applicants. Domains including faculty information, resident information, incentives, wellness, and environment are areas that require improvement and content updates. Addressing these insufficiencies by updating websites may help anesthesiology programs attract prospective applicants as part of their recruitment efforts and may help applicants make informed decisions surrounding their postgraduate training.
